# DELIVERING PERSON-CENTERED CARE IN TIMES OF CRISIS

**DOI:** 10.1093/geroni/igac059.917

**Published:** 2022-12-20

**Authors:** Liza Behrens, Tonya Roberts

## Abstract

Preference-based person-centered care (PCC) is the gold standard for delivering high quality care in nursing homes (NH). A public health crisis makes it difficult for NH leaders to make decisions that honor residents’ preferences for care and activities while balancing the safety of others in the NH community. Based on the Operational Framework to Guide Decision-making During the COVID-19 Pandemic, the purpose of this symposium is to discuss several projects that are growing the evidence-base for person-centered risk management during a public health crisis. Data presented can guide NHs in balancing safety and risk during a public health crisis. Our first presentation speaks to the adoption of preferences as an important measure of care quality prior to a crisis, the next two presentations speak to how risk perceptions of NH staff and residents can support PCC delivery during a crisis, and the final presentation provides a potential evidence-based quality improvement strategy for integrating PCC in the pandemic recovery phases. First Dr. Abbott will present on the impact of using the preferences for everyday living inventory as a quality and safety indicator in NHs, then Dr. Carpenter will present on direct-care staff members risk perceptions while during the COVID crisis, followed by Dr. Behrens who will discuss residents’ risk perceptions around having their preferences honored during the COVID crisis, and finally Ms. Heilbrunn will discuss an ongoing clinical trial evaluating the impact of using the ECHO platform to improve infection control and care quality during the COVID crisis.

